# Role of the Neurologic System in Fracture Healing: An Extensive Review

**DOI:** 10.1007/s11914-023-00844-0

**Published:** 2024-01-18

**Authors:** Reginald S. Parker, Murad K. Nazzal, Ashlyn J. Morris, Jill C. Fehrenbacher, Fletcher A. White, Melissa A. Kacena, Roman M. Natoli

**Affiliations:** 1 Department of Orthopaedic Surgery, Indiana University School of Medicine, Indianapolis, IN USA; 2 Department of Pharmacology and Toxicology, Indiana University School of Medicine, Indianapolis, IN USA; 3 Indiana Center for Musculoskeletal Health, Indiana University School of Medicine, Indianapolis, IN USA; 4 Stark Neuroscience Research Institute, Indiana University School of Medicine, Indianapolis, IN USA; 5 Department of Anesthesia, Indiana University School of Medicine, Indianapolis, IN USA; 6 Richard L. Roudebush VA Medical Center, Indianapolis, IN USA

**Keywords:** Neural regulation, Fracture healing, Autonomic nervous system, Neuropeptides, Pain management in fractures, Fracture and Neural Regulation, Fracture and Neuropeptides, Fracture and Traumatic Brain Injury, Fracture and Chronic Pain, AI, Artificial intelligence, ChatGPT

## Abstract

**Purpose of Review:**

Despite advances in orthopedics, there remains a need for therapeutics to hasten fracture healing. However, little focus is given to the role the nervous system plays in regulating fracture healing. This paucity of information has led to an incomplete understanding of fracture healing and has limited the development of fracture therapies that integrate the importance of the nervous system. This review seeks to illuminate the integral roles that the nervous system plays in fracture healing.

**Recent Findings:**

Preclinical studies explored several methodologies for ablating peripheral nerves to demonstrate ablation-induced deficits in fracture healing. Conversely, activation of peripheral nerves via the use of dorsal root ganglion electrical stimulation enhanced fracture healing via calcitonin gene related peptide (CGRP). Investigations into TLR-4, TrkB agonists, and nerve growth factor (NGF) expression provide valuable insights into molecular pathways influencing bone mesenchymal stem cells and fracture repair. Finally, there is continued research into the connections between pain and fracture healing with findings suggesting that anti-NGF may be able to block pain without affecting healing.

**Summary:**

This review underscores the critical roles of the central nervous system (CNS), peripheral nervous system (PNS), and autonomic nervous system (ANS) in fracture healing, emphasizing their influence on bone cells, neuropeptide release, and endochondral ossification. The use of TBI models contributes to understanding neural regulation, though the complex influence of TBI on fracture healing requires further exploration. The review concludes by addressing the neural connection to fracture pain. This review article is part of a series of multiple manuscripts designed to determine the utility of using artificial intelligence for writing scientific reviews.

## Introduction

This is one of many articles evaluating the utility of using AI to write scientific review articles on musculoskeletal topics [1]. The first draft of this review was written by humans and ChatGPT4.0 whereby humans selected literature references, but ChatGPT 4.0 completed the writing. Importantly, the article was edited and carefully checked for accuracy resulting in a final manuscript which was significantly different from the original draft. Refer to this edition’s Comment paper for more information [2]. The incidence of delayed union and nonunion following long bone fractures emphasizes the urgent need for improved understanding and management of fracture healing [3]. Recent studies have highlighted the role of certain bone marrow cells as critical components of fracture healing [4]. However, bone repair is influenced by many other factors including the nervous system. The complex relationship between the nervous and skeletal systems extends beyond the structural and mechanical interactions, delving into complex biochemical and cellular interactions that significantly influence the bone healing process [5, 6].

The healing of long bone fractures by endochondral ossification is a complex procedure encompassing three intertwined stages: a reactive stage, a reparative stage, and a remodeling stage. The initial response, which peaks between 24 and 48 h after the injury, results in hematoma formation and the gathering of inflammatory cells and fibroblasts to produce granulation tissue. The repair stage starts a few days post-fracture and can continue for weeks, during which versatile mesenchymal cells differentiate into fibroblasts, chondroblasts, or osteoblasts. The remodeling phase involves the replacement of the initial woven bone with lamellar bone, a process that can take several months to years [5]. The influence of neural mechanisms is vital in the process of fracture repair, serving not only as a controller of pain and swelling but also as a key factor in promoting bone recovery.

Despite abundant understanding of the roles of fibroblasts, chondroblasts, and osteoblasts on fracture healing, the exact mechanisms by which the nervous system contributes to fracture healing are not fully understood. Recent research has begun to shed light on these mechanisms, and by deepening our understanding of the neural regulation of fracture healing, we can develop more effective strategies to address common and costly fracture healing complications.

In this review, we will explore the roles of the peripheral and central nervous systems (PNS and CNS, respectively) in fracture healing, delving into the direct pathways linking these systems to bone healing. We will examine the impact of various molecular factors on healing and the relationship between neural regulation and fracture pain in traumatic injury models. We will evaluate clinical as well as preclinical research. The latter is particularly important to better understand the mechanisms of bone repair that may reveal novel therapeutic targets.

## Peripheral Nervous System Regulation of Fracture Healing

The PNS plays a vital role in the regulation of fracture healing, with more clearly defined effects compared to the CNS. Peripheral nerve stimulation and PNS-associated molecules are key components in the fracture healing process, contributing significantly to the orchestration of bone repair and regeneration. Recent research has shown the multifaceted relationship between nerves and bone, highlighting that trophic signals, protective pain, and the regulation of blood flow are all indispensable components of neural regulation of bone health [7]. This interplay between the PNS and bone health sets the stage for a deeper exploration into the specific effects that the PNS and associated molecular factors exert on the process of fracture healing.

The PNS which comprises sensory, autonomic, and motor nerves plays a pivotal role in fracture healing. This has been confirmed through various experimental animal models, including nerve resection models, which have provided valuable insights into the mechanisms of fracture healing. Individually, sensory and autonomic nerves have been identified as key factors that alter the healing process.

### Peripheral Reinnervation Following Fracture in Preclinical Animal Models

The intricate development of sensory and autonomic nerves in the skeletal system, as observed in embryonic and newborn mice, highlights the critical role of these nerve supplies in skeletal development, particularly in regions of high osteogenic activity, such as fracture sites [8].

The innervation of bone and reinnervation of fracture callus following injury have been well characterized using growth-associated protein-43 (GAP-43) and protein gene product 9.5 (PGP 9.5). GAP-43, a marker of new axonal growth and innervation, is highly expressed during the early stages of fracture healing, indicating active nerve regeneration or outgrowth, and is associated with nerve sprouting into the fracture callus before vascularization in rats [9]. GAP-43–positive nerve fibers persist in the periosteum, muscle, and callus of the fracture site after fully healing. This suggests that innervation is essential for bone growth and remodeling [9]. In addition, a time-dependent increase in the co-expression of GAP-43 and PGP-9.5, which tends to indicate only mature nerves, in the newly formed bone and connective tissue indicates the maturation of many nerves over the duration of fracture healing.

### Effects of Peripheral Denervation on Fracture Healing in Preclinical Animal Models

The proliferation of nerve fibers into the healing fracture callus is unsurprisingly attenuated by sciatic nerve resection, concomitant with significant impairment of fracture healing. The acceleration of rat tibial fracture union through the formation of bridging calluses is a common outcome of both spinal and sciatic denervation. However, these calluses, despite their rapid formation, are typically less dense and characterized by a reduced presence of collagenous matrix and minerals, suggestive of an osteoporotic transformation [10]. Radiographs support these findings, showing increased callus formation in fractured animals with sciatic nerve resection; however, there is a decrease in mechanical strength, suggesting a defective organization of the callus [11]. In contrast to primary fracture healing, which involves direct bone regeneration without a callus, secondary fracture healing is distinguished by the formation of a callus, followed by gradual bone remodeling. The PNS, particularly sensory innervation, plays a crucial role in recognizing movement in the fracture. In the presence of minimal movement, nerve signals initiate primary bone healing [11]. However, the resection of peripheral nerves changes the pattern of innervation of the fracture callus and adjacent bone, leading to a shift in the healing pattern [12, 13].

Periosteal stripping, which results in the removal of periosteal nerve terminals, also led to delays in osteotomy healing and the development of atrophic nonunions, which are fractures that fail to heal [14, 15]. Selective stripping of sensory neurons via local administration of excitotoxic capsaicin significantly affects fracture healing. Sensory denervation impacts the expression of collagen I, a major component of the extracellular matrix in bone, and influences the balance between bone formation and resorption during fracture healing. In bones with sensory denervation, the collagen fibrils in the fracture repair zone do not follow an organized neat pattern but are irregularly spaced throughout [16]. Moreover, a gradual reduction of collagen II along with the simultaneous increase of collagen I in the healing tissue at 2 weeks and 4 weeks post-fracture indicates a dynamic remodeling of the collagen matrix during the healing process [17]. Similarly to the callus trends seen in sciatic nerve resection, the fracture callus was found to be larger but less ossified in the sensory-denervated group than in the sensory-intact group, further highlighting the influence of sensory nerves on bone healing [16]. Altogether, these findings suggest that denervation of the fracture site, especially sensory denervation, compromises fracture healing.

### Effects of Peripheral Nerve Activation on Fracture Healing in Preclinical Animal Models

The importance of intact sensory innervation is further highlighted when considering therapeutic interventions like low-intensity pulsed ultrasound. Ultrasound treatment to the fracture site significantly boosts the rate of union and the volumetric bone mineral density (BMD) in the fracture callus of rats with intact neural pathways, but its effectiveness diminishes in the absence of sensory innervation [18].

Dorsal root ganglia (DRG) contain the soma for sensory neurons, and significantly influence various physiological processes, including fracture healing. Electrical stimulation (ES) at the DRG has been shown to effectively promote fracture healing by increasing calcitonin gene related peptide (CGRP) expression in both DRG and the fracture callus [19••], suggesting a potential approach for promoting healing of limb fractures [20]. Upregulation of CGRP contributes to type-H vessel formation, a biological event coupling angiogenesis and osteogenesis, thereby enhancing osteoporotic fracture healing [19••]. These findings suggest that sensory nerve activation directly improves the rate and quality of fracture healing.

Research has shown that osteoblasts respond rapidly to mechanical loading, upregulating certain signaling pathways within an hour of loading, and this effect is dependent upon TrkA-expressing sensory neurons [21]. This suggests that the effects observed in sciatic nerve resection models might be influenced by these early sensory neuron responses to mechanical stimuli, signifying that this is an alternative site of action for sensory neurons that should be explored further. These findings further underscore the intricate role of the PNS in fracture healing, shedding light on the complex interplay between nerve growth, stimulation, and the process of bone healing and remodeling.

### Autonomic Nervous System

The autonomic nervous system (ANS), encompassing the sympathetic (SNS) and parasympathetic nervous systems, also plays a pivotal role in bone metabolism and fracture healing. Study of the autonomic nervous system is wrought with complexity, as the sympathetic nervous system influences multiple aspects of physiology and pathology. Preclinical evaluation of the role of the sympathetic nervous system can be achieved by several methods. Systemic administration of 6-hydroxydopamine (6-OHDA) induces a peripheral chemical sympathectomy; thus, effects of 6-OHDA are wide ranging and include the immune system [22]. Alternatively, sympathetic nerve bundles can be transected to examine a more limited effect of sympathetic denervation on fracture healing. In preclinical models of fracture, systemic sympathectomy reduces the mechanical stability of the bone, suggesting a role in fracture healing and mechanical strength [22, 23]. It is important to note that the effects on mechanical stability may be due to the homeostatic effect of the SNS on BMD or via the regulation of immune responses to fracture.

Sympathetic denervation can also impact the maturity of new bone formation in distraction osteogenesis (DO) models, providing additional evidence for the importance of the SNS in bone regeneration [24]. In cases of trophic disturbances in the lower extremities, the SNS plays a crucial role, with the first sacral ganglion being particularly significant [25]. Moreover, the SNS can influence the growth rate of an extremity, as evidenced by the effects of a surgical sympathectomy. A successful lumbar sympathectomy performed in children with polio, which results in a sustained increase in circulation, can cause an acceleration of the rate of growth of a leg affected by conditions such as infantile paralysis [26]. This procedure can also halt the progression of shortness in the affected leg, suggesting that the SNS plays a role in the regulation of bone growth through regulation of blood flow [26].

The SNS also influences bone remodeling via release of norepinephrine (NE) and activation of cognate β2-adrenergic receptors on osteoblasts, cells responsible for bone formation. Activation of sympathetic signaling is associated with low BMD in humans and animal models [27, 28], and beta-blockers reverse this suggesting that beta-blockers might be used as bone anabolic and anti-catabolic drugs and that persistent exposure of osteoblasts to norepinephrine leads to decreased bone formation and increased bone resorption [29, 30]. Indeed, there are ongoing clinical trials investigating this possibility.

### Molecular Factors of the PNS

**Fig. 1:**
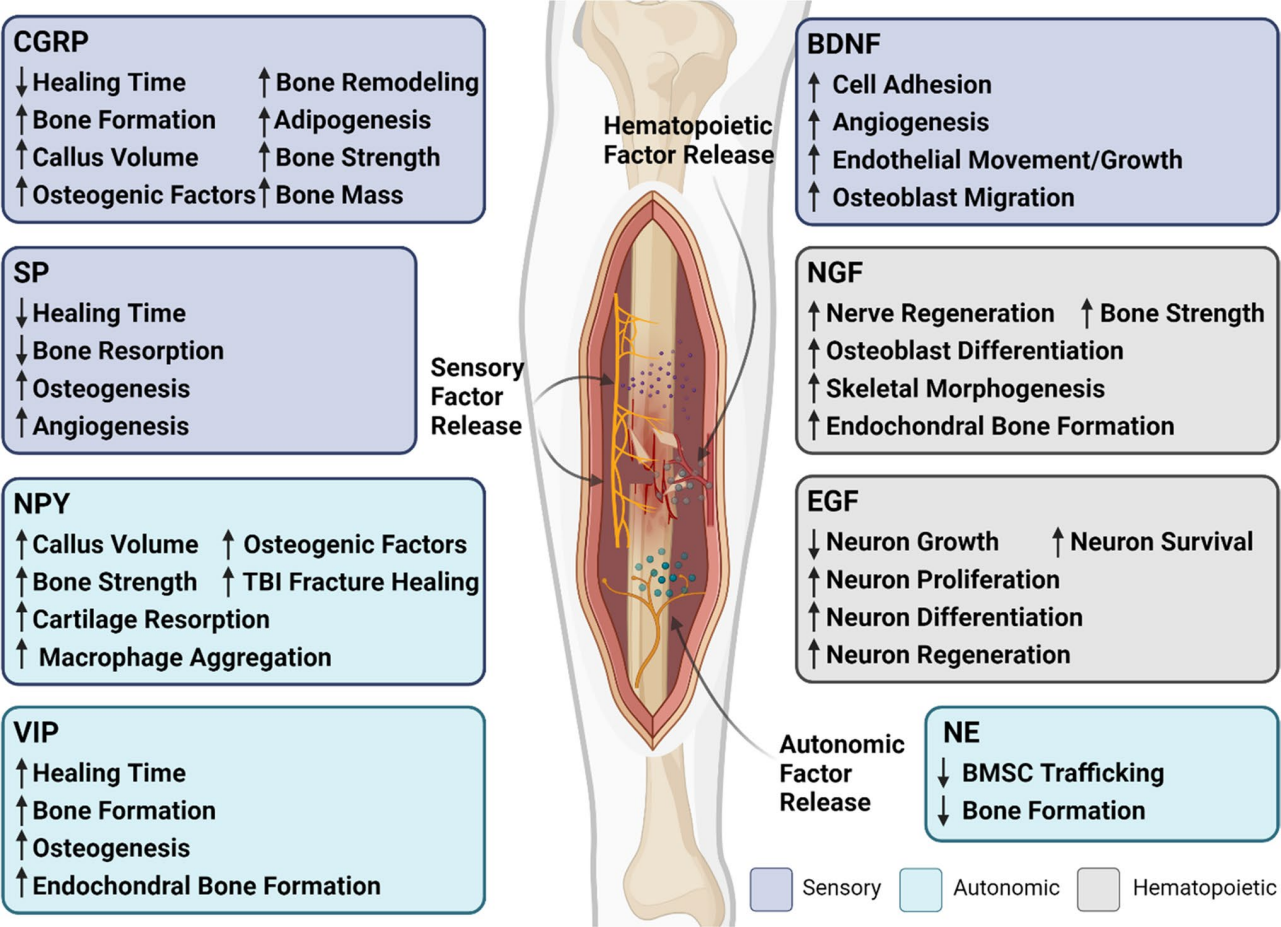
Neurotrophic and neuropeptide impact on fracture healing. This figure summarizes the roles of neurotransmitters and neurotrophic factors in bone repair, illustrating their diverse effects on healing pathways. CGRP, SP, NPY, and VIP are represented on the left, detailing their contributions to reduced healing time and enhanced bone formation and osteogenesis. The central image depicts a bone cross-section with sensory, autonomic, and hematopoietic-derived factor release, signifying the localized effects of these factors. To the right, BDNF, NGF, and EGF are linked with cellular processes essential for bone repair, such as cell adhesion, nerve regeneration, and osteoblast differentiation. The negative influences of NE on bone formation and bone marrow stem cell (BMSC) trafficking are also noted, demonstrating the complex regulatory mechanisms of neurotransmitters in fracture healing. Created with BioRender.com

Fracture healing is a complex process that involves a multitude of molecular factors and signaling pathways that originate from the nervous system. Recent research has highlighted the role of various neuropeptides and signaling molecules in this process, providing new insights into the mechanisms of bone regeneration and the potential for therapeutic interventions. Specifically, peptidergic neuropeptides such as CGRP, SP, and vasoactive-intestinal peptide (VIP) have been found to accelerate fracture healing. Neurotrophins like nerve growth factor (NGF) and brain-derived neurotrophic factor (BDNF) uniformly stimulate osteogenesis, thereby promoting fracture healing [31]. These findings highlight the intricate interplay between neural regulation and bone healing, opening new avenues for therapeutic strategies. Figure 1 shows the effects CGRP, SP, neuropeptide Y (NPY), VIP, BDNF, NGF, epidermal growth factor (EGF), and NE on fracture healing. These factors will all be discussed with respect to their roles in connecting the nervous system to fracture healing.

Neuropeptides released by sensory nerve fibers via axonal transport may exert paracrine trophic effects on bone, such as CGRP, which has been implicated in the regulation of bone remodeling [13]. CGRP is a neuropeptide distributed throughout the CNS and PNS, playing a significant role in processes like vasodilation, immune cell modulation, and pain transmission. Research indicates that CGRP serves as a pivotal orchestrator, interconnecting the neural, immunological, and skeletal systems throughout the process of bone repair [32]. In humans, an elevation of CGRP and SP in the serum of patients was noted with femur fractures [33]. In a rabbit mandibular injury model, CGRP expression was found to increase over a period of 14 days after injury, reaching its maximum at this point [34]. This timepoint coincided with the transition from the inflammatory stage to new bone regeneration in a rabbit bone-tendon interface injury model [35, 36]. These findings indicate that CGRP expression may correlate to the transition in stages of fracture healing in mammalian models. Moreover, CGRPα-deficient mice displayed impaired bone regeneration, suggesting the essential role of CGRPα in bone healing [37]. Comprehensive genomic studies reveal that CGRP stimulates specific genes related to ossification, bone remodeling, and adipogenesis, indicating a crucial role for CGRP receptor-dependent peroxisome proliferator-activated receptor gamma (PPARγ) signaling in fracture healing [37]. These data also suggests that CGRP may promote the proliferation of periosteal progenitor cells, crucial for new bone formation during fracture healing. Research has shown that inhibiting CGRP signaling by blocking receptor binding or deleting the receptor resulted in reduced callus volume, bone mass, and bone strength [38•]. Furthermore, CGRP was found to modulate M2 macrophages, influencing the secretion of osteogenic factors such as bone morphogenetic protein-2 (BMP-2), BMP-6, WNT10b, and oncostatin M [32]. Macrophages, which have been reported to play a functional role during bone development and regeneration, were found to aggregate in the fracture area during the healing process, suggesting a potential relationship between CGRP and macrophage localization during fracture repair, further underscoring the multifaceted role of CGRP in the healing process [39].

In addition to CGRP, SP has been implicated in aspects of bone healing, including angiogenesis. Models of fracture nonunion have revealed a substantial reduction in the mRNA expression of both CGRP and SP [40]. Both CGRP and SP are increased in the first 24 h after fracture [41]. Inhibition of the receptor for SP (neurokinin-1-tachykinin receptor) has been linked to decreased gene expression and biomechanical strength in bone healing, suggesting a potential therapeutic role for SP in disrupted fracture repair [42]. Mechanistically, SP was found to promote osteogenesis and mitigate bone resorption via activated Wnt signaling [43].

NPY also has been implicated in fracture healing, with several studies highlighting its significant role in this process. An increase in autonomic NPY-positive nerve fibers was observed using immunohistochemical staining at the fracture site, suggesting a significant role of NPY in fracture healing [44]. Acting via its Y1 receptor, NPY has been found to play a crucial role in bone repair. Global deletion of the Y1 receptor has been shown to hinder fracture healing. Specifically, in Y1 receptor-deficient mice, there is a delay in fracture repair, as seen by a decrease in bone callus volume and callus strength. Additionally, the histopathological characteristics revealed an increase in the avascular and cartilaginous regions, leading to a subsequent delay in cartilage resorption, thereby resulting in hindered bone union [45]. NPY has also been found to be associated with macrophage aggregation during the fracture repair process [39]. As mentioned above, macrophages play a significant role in the immune response and tissue repair, including bone regeneration. Altogether, these findings suggest that NPY may directly contribute to bone formation and may also indirectly facilitate healing by influencing the immune response [39].

BDNF plays a significant role in fracture healing, with high levels of BDNF and its receptor, TrkB, found at fracture sites [46]. BDNF and TrkB immunoreactivity and mRNA analyses show that the ligand is expressed predominantly by endothelial cells and the receptor is expressed on osteoblastic lineage cells within the fracture microenvironment [47]. BDNF promotes osteoblast migration and increases integrin β1 expression, a protein involved in cell adhesion and migration, through TrkB receptor activation of ERK1/2 and AKT signaling pathways [48]. While BDNF does not significantly affect the expression of receptor activator of nuclear factor kappa beta (RANKL) or osteoprotegerin (OPG), key regulators of osteoclastogenesis and bone resorption in MLO-Y4 osteocytes [49], it does increase OPG expression in earlier osteoblast progenitor MC3T3 cells in a TrkB-dependent manner [50]. BDNF also has been shown to enhance endothelial cell movement, growth, and the creation of new vasculature, primarily through the augmentation of vascular endothelial growth factor (VEGF) production [51]. Furthermore, BDNF administration has been found to elevate the levels of alkaline phosphatase (ALP) and BMP-2 in cementoblasts, cells that share similarities with osteoblasts [52]. Despite all of these putative bone-anabolic functions of BDNF, the TrkB agonist, 7,8-DHF, impaired fracture healing in mice, suggesting that TrkB activation and possibly BDNF’s downstream effects may have varying impacts on bone health and fracture healing [46].

NGF plays a significant role in bone fracture healing. In general, NGF promotes local regulation of nerves leading to increased neuropeptide expression, cytokine levels, and sensory innervation density to promote the bone healing process [53]. NGF is found to be expressed in cells such as osteoclasts, osteocytes, chondrocytes, synovial fibroblasts, and immune cells [54, 55]. NGF mediates osteoblast differentiation, a critical process in bone formation and fracture healing, via the BMP-2/Smad pathway. This pathway regulates the expression of runt-related transcription factor 2 (Runx2), a transcription factor crucial for osteoblast differentiation and skeletal morphogenesis. Observations indicate an elevation in Runx2 protein levels when exposed to NGF, implying a potential enhancement of the BMP-2/Smads/Runx2 signaling cascade by NGF [54]. NGF in the callus following a femur fracture was observed in aged mice to be increased relative to their younger counterparts, implying a heightened demand for neural restoration and skeletal restructuring in the elderly [56•]. Further, Li et al. [57] reported that NGF expression was upregulated in the fracture callus, peaking at day 3 post-fracture. NGF also plays a role in the transition from cartilage to bone during the healing process. Indeed, local injections of β-NGF resulted in a gene expression profile favoring endochondral bone formation and enhancing the structural integrity of the healing bone [58].

The neuropeptide VIP, released by the SNS, promotes in vitro osteogenesis of bone marrow mesenchymal stem cells (BMSCs) and in vivo bone defect repair [23]. The SNS also influences endochondral ossification, a crucial process in fracture healing. The absence of sympathetic neurotransmitters delays the differentiation of mesenchymal callus tissue toward a cartilaginous matrix, suggesting a role in callus formation and differentiation [22]. Fracture chondrocytes produce SP and its receptor NK1, indicating an endogenous callus signaling loop [59]. This suggests that sensory and sympathetic neurotransmitters play a crucial role in the regulation of callus formation and differentiation during fracture healing [22].

Sympathetic denervation has also been shown to reduce NE levels in DO, leading to a decrease in adrenergic receptor beta 3 (adrb3) in bone marrow stromal cells and promoting their migration to bone-forming areas [60]. While NE typically inhibits mesenchymal stem cell migration and osteogenic differentiation, this effect can be reversed by using siRNA to knockdown adrb3 [60].

In summary, the molecular factors influencing fracture healing are diverse and interconnected, involving various neuropeptides, neurotransmitters, and signaling pathways. Although further research is needed to fully elucidate their complex interactions with other biological systems, understanding these molecular mechanisms can provide valuable insights into the process of bone regeneration and offer potential targets for therapeutic interventions to enhance fracture healing.

## Central Nervous System Regulation

The role of the CNS in the regulation of fracture healing, though seemingly counterintuitive at first glance, has gained significant recognition in recent years. The last two decades have witnessed a surge in research illuminating direct pathways that link the CNS to bone healing processes. Traumatic injury models have served as valuable tools in establishing connections between the CNS and fracture healing, thereby opening new avenues for understanding the complex interplay between the nervous system and skeletal repair mechanisms.

### Traumatic Brain Injury Effect on Fracture Healing

Traumatic injuries to the CNS can have profound and multifaceted impacts on the body, extending beyond the immediate neurological implications. Many retrospective cohort studies have been conducted to examine the effects of TBI on fracture healing. TBI including its mild form (mTBI) can significantly impact bone health, leading to growth hormone deficiency and disrupted bone mass accrual, which increases the risk of fractures and osteoporosis [61, 62]. TBI can also induce sympathetic hyperactivity, further affecting bone health [63]. Clinical studies have shown a relationship between TBI and bone-related abnormalities such as osteoporosis, osteopenia, and increased risk of fractures [62]. Regardless of mobility restrictions placed on animals, TBI notably diminishes cortical bone and leads to a reduction in the mass of trabecular bone in the tibia of murine models [62, 64]. Osteoporosis treatments like bisphosphonates can help reverse bone damage associated with inflammation-related bone loss [65]. Therefore, understanding these mechanisms and strategies to mitigate TBI’s secondary skeletal effects can improve patient outcomes.

The interplay between TBI and bone fracture healing presents a complex picture. Preclinical research suggests that TBI may accelerate callus formation and alter its composition and strength, as evidenced by mice with TBI exhibiting earlier bridging callus formation and greater final mean callus thickness than those without TBI [66]. This acceleration may be due to the release of osteoinductive factors, such as BMPs [62], into the systemic blood circulation and increased hematoma formation associated with TBI in patients [67–69]. The effect of TBI on fracture healing also may be site-specific, as the association of head injury did not increase the rate of union in forearm fractures [70]. Clinically, rapid union of fractures in association with severe head injury may lead to malunion, particularly in patients where treatment of cerebral and other injuries takes priority over their fractures [71]. The quantity of callus formation in fracture patients who have sustained head injuries notably surpasses that observed in a control (no head trauma) group, implying a direct influence of the head trauma on the fracture healing trajectory [72]. The interpretation of these head trauma effects as either an acceleration in fracture healing or a manifestation of local heterotopic ossification (HO) continues to be unclear [73, 74]. Of note, there are important caveats to consider. Specifically, patients sustaining a TBI and femoral fracture who were treated non-operatively did not develop excessive callus formation [75]. Furthermore, when TBI is of a repeated mild nature (r-mTBI), it may impair the healing process, leading to a significant reduction in the bone volume/tissue volume (BV/TV) phenotype and a decrease in the mechanical strength of the newly formed bone in mice [76]. These contrasting findings underscore the complexity of TBI’s impact on fracture healing, which may be influenced by the severity and frequency of the injury.

Indeed, the severity of TBI, as measured by the Glasgow Coma Scale (GCS), has been found to correlate with the volume of callus formed [77]; however, clinical correlations are inconsistent between elements like the intensity of the cerebral trauma, the nature of the intracranial bleed, and patient sex with the pace of bone recuperation or the magnitude of ultimate callus development [78]. Humoral factors, including growth hormone, IL-6, and prolactin, are elevated in patients with TBI and may contribute to enhanced osteogenesis and more pronounced callus formation [77]. These factors could be potential targets for therapeutic interventions to enhance fracture healing in patients with TBI.

HO is a negative side effect observed in high prevalence following TBI [79]. HO is a pathological process characterized by the formation of ectopic bone in non-skeletal tissues, including muscles, tendons, and other soft tissues. This aberrant bone formation can occur in response to various stimuli, including trauma, neurological injury, specific genetic disorders, or as a side effect of certain surgical procedures. Management of HO aims to limit its progression and maximize joint function, with surgical excision considered in severe cases [80]. The complex interplay between hyperadrenergic activity, HO, and fracture healing following TBI or draws attention to the need for further research to develop effective management strategies [62, 81–83]. The management of complications such as HO also remains a challenge.

Animal models of combined TBI and standard closed fracture have demonstrated a systemic response that enhances fracture healing, particularly through increased stiffness and the stimulation of mesenchymal stem cell proliferation in rats [84]. These models, which may mirror high-energy trauma scenarios, provide a platform for exploring the interplay between neuronal, endocrine, and metabolic consequences of TBI and the individual fracture healing response [84]. Overall, while compelling evidence suggests that TBI may influence fracture healing, affecting callus volume, composition, and strength, further research is needed to elucidate the precise mechanisms underlying this phenomenon in patients with TBI.

### Molecular Aspects of TBI in Animal Models

Although the effects of TBI on fracture healing are inconsistent, it is still informative to highlight several signaling molecules that are affected by TBI that have fracture-healing effects. NPY contributes to post-fracture bone healing, especially in patients with TBI-fracture combined injuries. NPY levels were found to be increased in patients with combined injuries, accompanied by a rise in bone healing markers such as ALP, osteocalcin, type I procollagen peptide, and carboxy-terminal telopeptide of type I collagen compared to those with fractures only [85••].

Studies have shown that when a fracture is combined with peripheral nerve injury, the healing process can be slowed down [86]. In contrast, a fracture combined with cerebral cortex injury will accelerate the healing process. The concentration of CGRP in the DRG varied depending on the site of injury [86]: spinal or cerebral cortex injuries increased CGRP and positively affected fracture healing. Additionally, research has demonstrated that CGRP augments the process of DO through the amplification of angiogenesis, which in turn facilitates the healing of bones [87]. As may be expected, CGRP expression significantly escalates near the fracture location in the group with both TBI and fractures [88, 89].

EGF has been identified as a critical factor in neuronal differentiation and its potential implications for bone healing are noteworthy. EGF has been found to be closely related to cell growth, proliferation, differentiation, and regeneration of the CNS. EGF may promote the formation of bone tissue and accelerate the synthesis and deposition of the matrix in patients with TBI and limb fracture [90]. Moreover, EGF can act as a neurotrophic factor in the CNS, promoting neurite outgrowth and cell survival of cultured cerebral, subneocortical telencephalic, glial cells, and cerebellar neurons [91].

NGF plays a critical role in fracture healing, particularly in the context of TBI. As detailed earlier, NGF is essential for the survival and development of central and peripheral neurons. Its synthesis and release increase following TBI or bone injury, leading to elevated serum NGF levels. This elevation is particularly noticeable in patients who have experienced TBI in conjunction with limb fractures, implying a crucial role of NGF in facilitating the healing of fractures in these individuals [90, 92]. NGF’s role in TBI and fracture healing is significant, promoting neuron development and enhancing osteogenic capacity, potentially accelerating the fracture healing process.

Together, the intricate interplay between neuropeptides and other growth factors plays a pivotal role in the process of fracture healing associated with CNS injuries. Understanding these mechanisms can provide valuable insights into the development of therapeutic strategies for enhancing fracture healing. However, further research is needed to fully elucidate the complex series of signals from hormones, growth factors, and mechanical and neuronal changes that lead to this clinical phenomenon.

## Neural Connections to Acute and Chronic Fracture Pain

Earlier, the important role of neuropeptides and neural regulation in bone fracture healing were discussed. Here we also examine the important role of the nervous system in fracture-associated preclinical pain behaviors or clinical pain. Neuropeptides such as SP and CGRP contribute to glial activation and nociceptive sensitization after a fracture, a state of hyperreactivity to painful stimuli. This process, involving a wide array of markers and receptors, underscores the intricacy of the molecular mechanisms at play [93]. Intense post-fracture nociceptive signaling induces neuropeptide release in the spinal cord, resulting in amplification of spinal neuropeptide signaling and inflammatory mediator expression [94]. Inflammation plays a role in promoting the growth of nerve fibers, indicating a link between the inflammatory response and neural regulation in the context of fracture healing [95], and highlighting the conundrum of how to treat fracture patients, as pain management strategies can significantly influence the healing process and long-term functional outcomes of fractures [96]. Treatment of fracture patients could be a trade-off between timely healing or the development of chronic pain, as the intensity of pain experienced at the onset can forecast the emergence of chronic pain. Thus, prompt pain control could decrease the likelihood of chronic pain development, but also delay healing [97]. Pain assessment methods used in animal models, such as assessing spontaneous and evoked pain behaviors, have clinical correlates in human patients [98, 99].

Chronic post-surgical/post-traumatic pain 1 year after surgery significantly impacts patients’ quality of life and mental health, with patients that develop chronic pain having lower health-related quality of life and higher levels of anxiety and depression compared to those without pain [100]. These findings highlight the need for effective interventions to prevent and manage chronic post-fracture pain.

The phenomenon of exuberant sprouting of sensory and sympathetic nerve fibers in non-healed fractures contributes to the generation and maintenance of chronic skeletal pain. The occurrence of nerve sprouting could potentially transform the perception of ordinarily non-painful skeletal loading into a noxious sensation, thereby possibly instigating a state of neuropathic pain [101]. Research using a femoral fracture mouse model has shown that an anti-NGF therapy could be considered to reduce pain after fracture surgery without affecting bone repair [102]. The importance of NGF in fracture healing has been highlighted earlier in this review. However, this study’s results show surprising evidence that anti-NGF therapy had no effect on fracture healing. The study measured fracture healing using μCT 6 weeks after surgery and saw no difference compared to controls. However, this data was not shown and may require further investigation. The understanding of these pain mechanisms could significantly enhance our ability to design effective and safe therapies for fracture pain, potentially reducing the reliance on opiates [103].

Fracture nonunion presents particular challenges. With nonhealed fractures (i.e., nonunions), chronic pain and altered gait/limping have been observed, suggesting a link between neural regulation and the chronic effects of fractures [96]. An increased expression of CGRP in neuronal soma and nerve proliferation into the scar tissue are evident in nonunion, resulting in persistent pain, and underscoring the importance of early achievement of bone union to minimize the neural changes causing persistent pain after fracture [95]. Impaired bone healing following fracture is also associated with a dysregulated long-term immune response in human fracture nonunion [104]. Non-responders (rats with defective critical size defect healing in response to BMP-2) demonstrate higher overall expression of inflammatory cytokines, including TNFα and IL-1β, compared to responders. The findings imply that the restoration of immune balance could be a pivotal factor in facilitating effective bone repair, especially in the context of nonunion scenarios [105]. The autonomic nervous system also plays a role in regulating adaptive immune system activation and nociceptive sensitization; thus, modulating autonomic activity post limb injury, either through suppression of sympathetic signals or enhancement of parasympathetic cues, may mitigate the immunological alterations associated with nociception following a fracture, thereby indicating a potential avenue to explore to reduce chronic pain [106••].

These findings mark the importance of understanding the role of neuropeptide signaling and inflammatory mediator expression in the context of fracture pain, both acute and chronic. That said, further research is needed to fully understand these complex interactions and their implications for the management of fracture pain and the promotion of bone healing, which could lead to the development of more effective therapeutic strategies.

## Conclusion

**Fig. 2:**
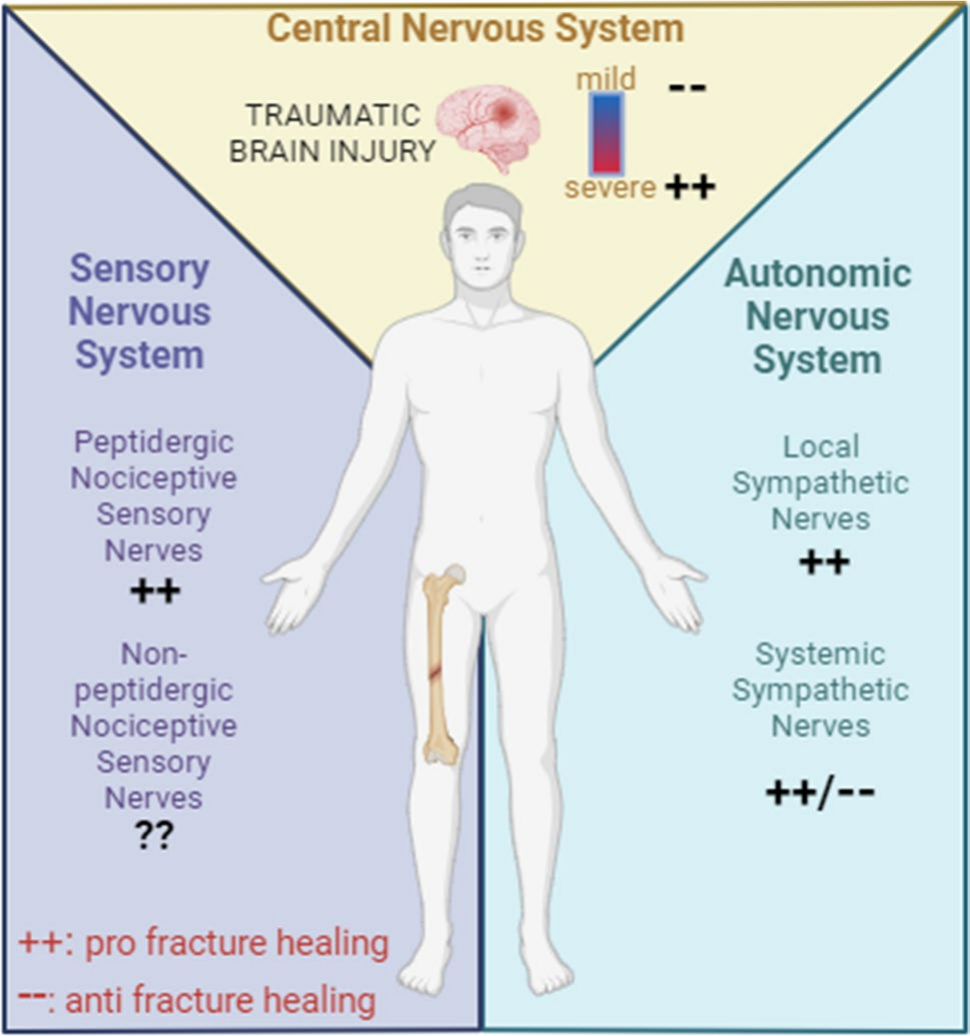
Nervous system regulation of fracture healing. This diagram delineates the influence of different components of the nervous system on fracture healing. The central nervous system (CNS) is represented at the top, with the effects of traumatic brain injury (TBI) on healing shown through varying degrees of impact. The sensory nervous system (SNS) is illustrated on the left with peptidergic or non-peptidergic nociceptive sensory fibers and their effect on fracture healing. The autonomic nervous system (ANS), divided into local sympathetic and systemic nerves, is mapped out on the right, indicating their respective roles in bone repair. Symbols and color gradients provide visual cues to their contributory or detrimental effects, with “ +  + ” denoting a positive influence and “—” a negative one on fracture healing. Created with BioRender.com

In conclusion, the neural regulation of fracture healing is a complicated and multifaceted process that involves the PNS and CNS, as well as a multitude of molecular factors. Peripheral sensory nerves, through release of neuropeptides, significantly enhance the healing process. The PNS influences bone cells, modulates the release of neuropeptides and neurotransmitters, and regulates endochondral ossification. The CNS, particularly in the context of TBI, can accelerate and impair the healing process. The role of various neuropeptides and growth factors, such as NPY, CGRP, EGF, and NGF, is pivotal in these processes. These molecules contribute to the regulation of bone homeostasis, angiogenesis, neuronal differentiation, and the growth and development of neurons, thereby influencing the fracture healing process. The complex interaction between the skeletal and nervous system is illustrated in Fig. 2.

The intricate interplay between these systems and factors highlights the complexity of the fracture healing process. However, it also presents potential therapeutic implications for enhancing fracture healing and managing fracture pain. Despite the significant progress made in understanding the neural regulation of fracture healing, there remain gaps in our knowledge. By deepening our understanding of these mechanisms, we can develop more effective strategies to address the common and costly complications associated with fracture healing, such as delayed union, nonunion, and post-traumatic fracture pain. In the future, the development of targeted therapies that can stimulate healing while attenuating pain could significantly improve patient outcomes following fractures.
